# The pivotal role of protein acetylation in linking glucose and fatty acid metabolism to β-cell function

**DOI:** 10.1038/s41419-019-1349-z

**Published:** 2019-01-25

**Authors:** Yuqing Zhang, Feiye Zhou, Mengyao Bai, Yun Liu, Linlin Zhang, Qin Zhu, Yufang Bi, Guang Ning, Libin Zhou, Xiao Wang

**Affiliations:** 1 Department of Endocrine and Metabolic Diseases, Shanghai Clinical Center for Endocrine and Metabolic Diseases, Shanghai Institute of Endocrine and Metabolic Diseases, Ruijin Hospital, Shanghai Jiaotong University School of Medicine, Shanghai, 200025 China; 2 Center for Reproductive Medicine, Shandong University, Jinan, 250000 China; 3 Key Laboratory of Reproductive Endocrinology, Ministry of Education, Shandong University, Jinan, 250000 China

## Abstract

Protein acetylation has a crucial role in energy metabolism. Here we performed the first large-scale profiling of acetylome in rat islets, showing that almost all enzymes in core metabolic pathways related to insulin secretion were acetylated. Label-free quantitative acetylome of islets in response to high glucose revealed hyperacetylation of enzymes involved in fatty acid β-oxidation (FAO), including trifunctional enzyme subunit alpha (ECHA). Acetylation decreased the protein stability of ECHA and its ability to promote FAO. The overexpression of SIRT3, a major mitochondrial deacetylase, prevented the degradation of ECHA via decreasing its acetylation level in β-cells. SIRT3 expression was upregulated in rat islets upon exposure to low glucose or fasting. SIRT3 overexpression in islets markedly decreased palmitate-potentiated insulin secretion, whereas islets from SIRT3 knockout mice secreted more insulin, with an opposite action on FAO. ECHA overexpression partially reversed SIRT3 deficiency-elicited insulin hypersecretion. Our study highlights the potential role of protein acetylation in insulin secretion.

## Introduction

Pancreatic β-cells display a great degree of plasticity to secrete insulin in response to nutrient availability^1,2^. Although many metabolic coupling factors have been proposed to modulate metabolic networks involved in fuel-induced insulin secretion, the enormous complexity of metabolism-triggered signaling processes is beyond our understanding^3^. The growing landscape of protein posttranslational modification (PTM) has highlighted its regulatory roles in cellular metabolism^4^. Therefore, application of large-scale proteomics should help us comprehensively understand the mechanism for islet β-cells to adapt to metabolic changes and provide insights into the pathogenesis of type 2 diabetes.

Protein lysine acetylation (Kac) is a conserved PTM that is emerging as a crucial regulator of protein function^5,6^. Recent advances in mass spectrometry have led to the identification of thousands of acetylated proteins^7–11^, highlighting the regulatory potential of acetylation in many biological processes. Acetylation level is tightly governed by lysine acetyltransferases (KATs) and deacetylases (KDACs)^12^. All KATs require acetyl-CoA as substrate for acetylation reactions. Another intermediary metabolite NAD^+^ directly alters KDAC activities to link energy status to cellular homeostasis, making acetylation especially favorable in regulating metabolic enzymes.

As fuel sensors, β-cells are extremely sensitive to nutrients alterations. The primary stimulus for insulin secretion is glucose, whose metabolism is achieved by tightly linking glycolysis with mitochondrial metabolism^13^. Fatty acids also have enormous capacity to amplify glucose-stimulated insulin secretion (GSIS), in part via their metabolism into lipid signaling molecules^14^. Given that β-cell function is closely coupled to fuel metabolism and protein acetylation may be at the nexus of coordinating metabolic flux, it is reasonable to hypothesize that protein acetylation may provide a link between fuel metabolism and insulin secretion. It has been shown that inhibition of class I histone deacetylases (HDACs) prevents cytokine-induced toxicity in β-cells^15,16^. The class III HDACs, sirtuins, also have important roles in insulin secretion^17–20^. These studies implicate the involvement of acetylation in regulating islet function. The substantial differences of acetylation patterns across tissues underly the importance of tissue-specific acetylome mapping^9^. However, the scope and targets of protein acetylation in islets remain largely unknown.

Here we report the first proteomic analysis of lysine acetylation in rat islets using affinity enrichment and high-resolution liquid chromatography tandem mass spectrometer (LC-MS/MS). Further quantitative acetylome of islets in response to high glucose revealed a critical role of acetylation in fatty acid oxidation (FAO) enzymes, among which trifunctional enzyme subunit alpha (ECHA, coding gene *HADHA*) and its deacetylase SIRT3 inhibited palmitate-stimulated insulin secretion via increasing FAO. This current study establishes protein acetylation as a link of glucose and fatty acid metabolism to adaptive islet β-cell function.

## Materials and methods

### Islet preparation, MS, and data analysis

Pancreatic islets were isolated from 8- to 12-week-old wild-type Sprague–Dawley (SD) male rats (Shanghai Laboratory Animal Company, Shanghai, China) by collagenase digestion and density-gradient centrifugation. For label-free sample preparation, isolated islets were incubated with either 3.3 mM (low) or 16.7 mM (high) glucose for 6 h in KRB buffer. Freshly isolated or incubated islets were washed twice with glucose-free phosphate buffer and were snap-frozen in liquid nitrogen before proteomic analyses. Islets from 25 rats were pooled to create a uniform sample of 20,000 total islets. Label-free samples were collected from three independent biological experiments. Mass spectrometry and data analysis were performed as described^21^ (see Supplementary Materials data for details). The quantitative proteomic method was label-free quantification (LFQ) and minimum score for modified peptides score was set ≥ 40. The acetylome datasets generated and analysed during this current study are available in the PRIDE repository, https://www.ebi.ac.uk/pride/archive/login. Username: reviewer56234@ebi.ac.uk Password: LiDVJTfM

### Insulin secretion assay

Isolated islets were cultured with indicated reagents in RPMI 1640 medium (0.25% bovine serum albumin (BSA)). To stimulate insulin secretion, islets were preincubated in Krebs-Ringer Buffer (KRB) containing 3.3 mM glucose for 30 min. Then, ten islets per assay in triplicate were incubated with KRB buffer containing either 3.3 mM glucose, 16.7 mM glucose, or other reagents as indicated for 1 h at 37 °C. Supernatants containing insulin were removed and stored at − 20 °C until analysis. Insulin content was extracted with acid-ethanol. Insulin levels of all samples were measured by ELISA kit (Mercodia).

### Animal studies

Breeding pairs of SIRT3 knockout mice (original 129/Sv background, purchased from Jackson Laboratories) were a generous gift from Dr. Weili Shen (Shanghai Key Laboratory of Hypertension, Department of Hypertension, Ruijin Hospital, Shanghai Jiaotong University School of Medicine) and have been backcrossed for at least five generations onto the C57BL/6 background (Shanghai Laboratory Animal Company, Shanghai, China). Ten- to 12-week-old male SIRT3KO mice and wild-type (C57BL/6) littermate controls on a standard chow diet were used. For fasting studies, SD rats were transferred to a new cage without food for 24 h.

### Cell culture and treatment

INS-1 cells were cultured in RPMI 1640 medium with 11.1 mM glucose that contained 10% fetal bovine serum (FBS). HEK293T cells were cultured in Dulbecco’s modified Eagle’s medium with 10% FBS. Cells were treated with 200 nM trichostatin A (TSA; Cell Signaling Technology) for 20 h, 5 mM nicotinamide (NAM; Sigma) for 6 h, or 10 μM MG132 (Sigma) for 6 h in the presence of 5.6 mM glucose. Plasmid transfection was carried out by Lipofectamine 2000 (Invitrogen).

### Western blotting and immunoprecipitation

Islets or INS-1 cells were collected in lysis buffer (Cell Signaling Technology). Protein lysates were separated by SDS-polyacrylamide gel electrophoresis and transferred to polyvinylidene difluoride membranes (BioRad). Primary antibodies were detected with horseradish peroxidase (HRP)-conjugated secondary antibodies. Anti-ECHA and anti-SIRT4 was from Abcam. Anti-SIRT3, anti-SIRT5, and anti-rabbit IgG conjugated with HRP were from Cell Signaling Technology. Anti-α-tubulin was from Proteintech. Anti-Flag was from Sigma-Aldrich. Anti-acetyllysine was from PTM Biolab. Immunoprecipitation was performed by incubating protein lysates with FLAG M2 Affinity Gel (Sigma) or ECHA antibody for 2 h and then with Protein A/G PLUS-Agarose (Santa-Cruz) overnight at 4 °C. The binding complexes were washed and then eluted with loading buffer. Standard western blotting was followed using anti-acetyllysine antibody for acetylation analysis.

### RNA isolation and qRT-PCR

Islet RNAs were extracted using RNeasy Plus Mini kit (Qiagen). Quantitative reverse transcription PCR (qRT-PCR) was performed with SYBR Premix Ex Taq (Takara) on a Light-Cycler 480 instrument (Roche Applied Science). Primer sequences are as follows: 18S (Forward: 5′-CACGGGTGACGGGGAATCAG-3′ and Reverse: 5′-CGGGTCGGGAGTGGGTAATTTG-3′); SIRT3 (Forward: 5′-GGCACTACAGGCCCAATGTC-3′ and Reverse: 5′-TCTCTCAAGCCCGTCGATGT-3′); SIRT4 (Forward: 5′-TTACAGCGCTTCATTAGCCTTTC-3′ and Reverse: 5′-CCCACCTTTTCTGACCTGTAGTCT-3′); SIRT5 (Forward: 5′-AGAGCAAGATCTGCCTCACCAT-3′ and Reverse: 5′-AGCCCCCGAGATGATGACTAT-3′);

### Adenovirus infection

For SIRT3 and ECHA overexpression, adenoviruses expressing rat target protein were constructed with a full-length target gene coding sequence. Islets or INS-1 cells were infected with target protein or vector adenovirus according to the manufacturer’s instructions (GeneChem).

### Oxygen consumption rate measurement

For FAO assay, except for the indicated treatment, islets were incubated in substrate-limited medium (RPMI 1640 medium with 0.5 mM glucose). Before the assay, islets were washed two times and then transferred to the Islet Capture Microplate (Seahorse Bioscience) with FAO assay medium containing 2 mM glucose. Oxygen consumption rate (OCR) was measured after XF Palmitate–BSA substrate was injected into the wells using a Seahorse XF24 flux analyzer (Seahorse Bioscience). For glucose oxidation assay, 20 mM glucose was preloaded into cartridges and injected into XF wells.

For FAO mitochondrial stress test, HEK293T cells were seeded in XF cell culture microplates and incubated in substrate-limited medium for 24 h after plasmid transfection. Cells were washed with FAO assay medium (supplemented with 2 mM glucose) and XF palmitate–BSA substrate or BSA was added to the wells just before starting the assay. The chemicals (1 μM oligomycin, 2 μM FCCP, and 0.5 μM rotenone/antimycin A) were preloaded into cartridges and injected into XF wells in succession. OCR was measured using Seahorse XF24 flux analyzer. Basal/maximal respiration due to utilization of exogenous palmitate was calculated as basal/maximal palmitate rate minus basal/maximal BSA rate.

### Statistical analysis

Unless otherwise indicated, all values are expressed as mean ± SEM and statistical significance was assessed by Student’s *t*-test. Differences were considered to be statistically significant when *p* < 0.05.

## Results

### Impact of HDAC inhibition on insulin secretion in rat islets

**Fig. 1:**
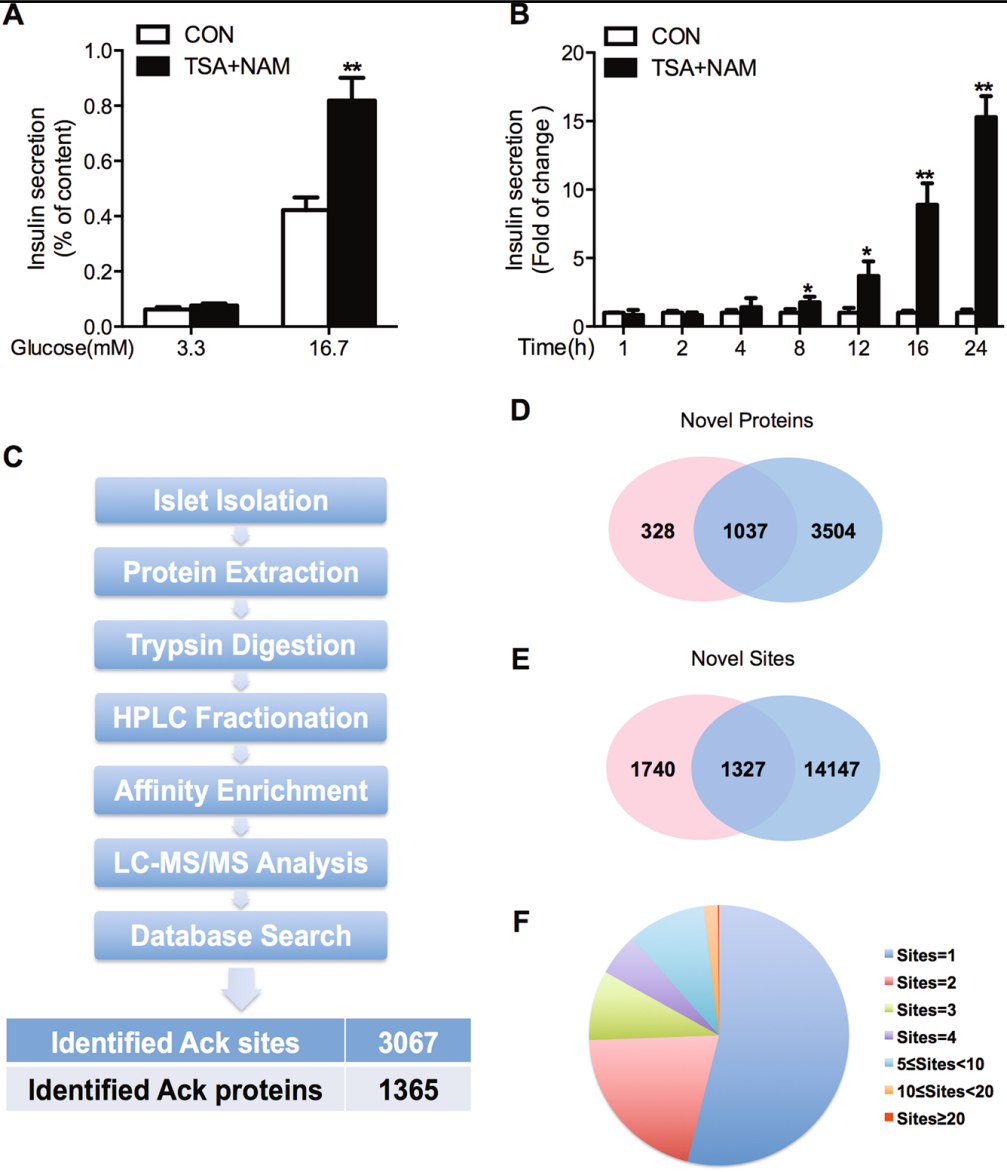
Profiling lysine acetylation proteome in rat islets. **a** Rat islets were stimulated with 3.3 or 16.7 mM glucose in the presence or absence of 200 nM TSA and 5 mM NAM for 1 h, and then insulin secretion was measured. **b** Rat islets were treated with 200 nM TSA and 5 mM NAM at 3.3 mM glucose for the indicated time and then insulin secretion was measured. **c** Proteomic workflow for the identification of acetylated sites and proteins in rat islets. **d** Comparison of the acetylated proteins identified in the present study (red circle) with that in CPLM database (blue circle). **e** Comparison of the acetylated lysine sites identified in the present study (red circle) with that in CPLM database (blue circle). **f** Distribution of the number of acetylated sites per protein. *p < 0.05, **p < 0.01 vs. control (CON)

To directly evaluate the role of protein acetylation in islet function, we investigated the combined effect of two HDAC inhibitors, TSA (inhibitor of HDAC I and II), and NAM (inhibitor of sirtuins), on insulin secretion. TSA and NAM co-treatment acutely enhanced GSIS in rat islets (Fig. 1a). In addition, insulin secretion in the presence of 3.3 mM glucose was markedly elicited with prolonged treatment of TSA and NAM (Fig. 1b). These data suggest that overall hyperacetylation promotes insulin secretion.

### Identification of acetylated sites and proteins in rat islets

To identify the acetylome in islets, we employed an integrated acetyl-proteomic approach for large-scale profiling. Primary islets from SD rats were isolated and pooled to account for biological variation. Protein extracts were enriched for lysine-acetylated peptides by immunoprecipitation and analyzed by LC-MS/MS. After searching against UniProt_rat database using MaxQuant, we identified 3067 lysine-acetylated sites in 1365 islet proteins (Fig. 1c and supplemental Table S1), among which 328 proteins and 1740 sites were never retrieved (Fig. 1d, e) in the CPLM database for lysine acetylation of rat proteins^22^. Evaluation of the high-quality MS data was shown in Figure S1A and S1B. We further calculated the number of acetylation sites per protein in this islet acetylome. Fifty-four percent of the identified proteins contained only one acetylation site, whereas 46% were multiacetylated (Fig. 1f).

### Characterization of the islet acetylation proteome

**Fig. 2:**
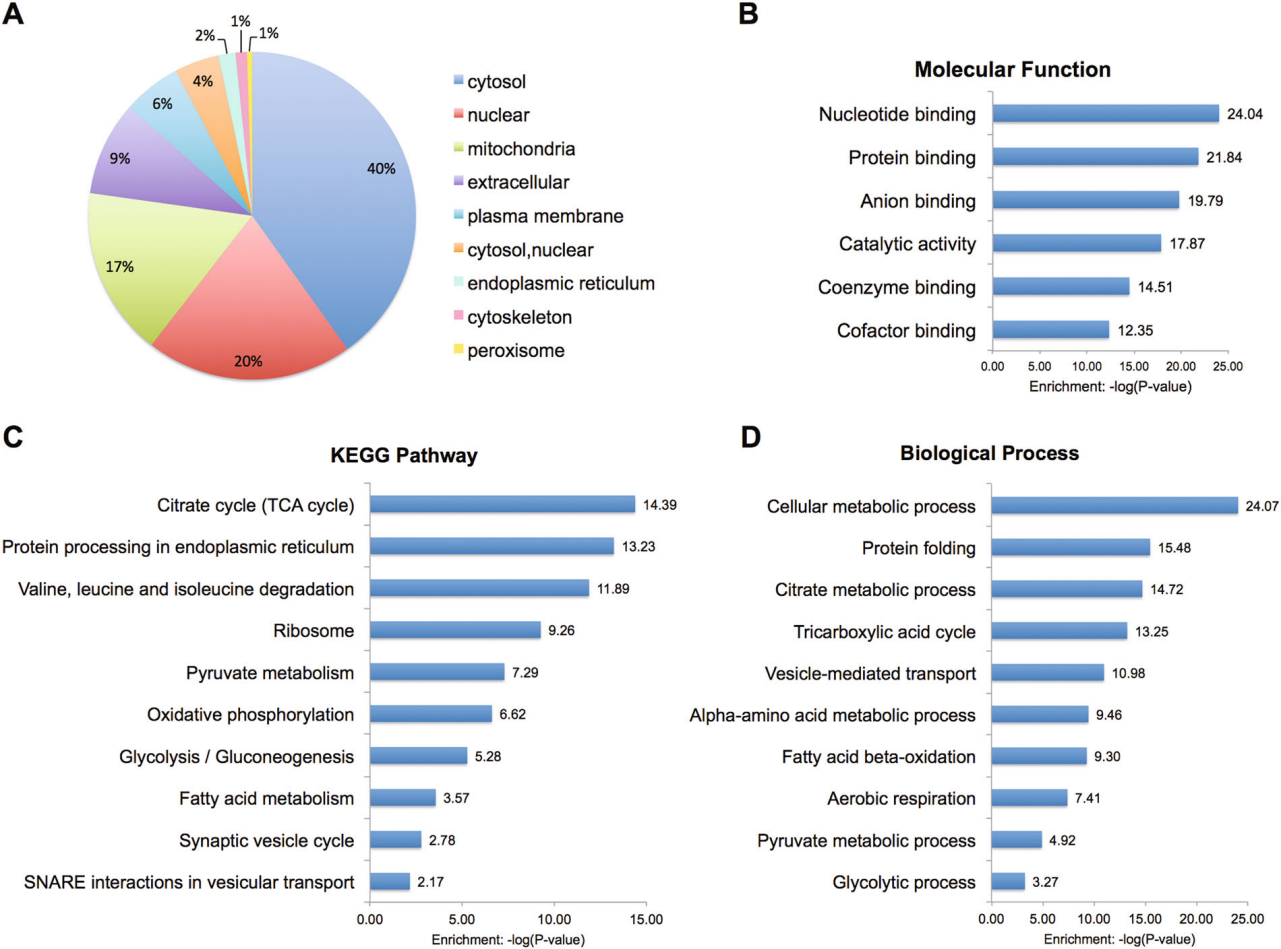
Characterization of the islet acetylation proteome. **a** Subcellular distribution of acetylated proteins identified in islets. **b** Representative GO molecular function term enrichment analysis of acetylated proteins. **c** Representative KEGG pathways enriched with islet acetylome. **d** Representative GO biological processes enriched with islet acetylome. Logarithmized corrected *p*-values for significant overrepresentation are shown

Subcellular distribution of islet acetylated proteins showed that the most abundant localization was cytosol. Proteins with exclusively mitochondrial annotation were also highly represented (Fig. 2a). Further analysis of molecular functions showed that the acetylated proteins were significantly enriched in binding and catalytic activities (Fig. 2b).

To understand islet-specific functions of the acetylated proteins, we performed the Kyoto Encyclopedia of Genes and Genomes (KEGG) pathway enrichment analysis. In agreement with acetylation-modulated cellular metabolism in the liver^11^, our data demonstrated that islet acetylated proteins were significantly enriched in metabolic pathways (Fig. 2c). Moreover, there was a significant enrichment for endoplasmic reticulum, ribosome, and vesicle transport pathways (Fig. 2c), implicating a regulatory role of acetylation in insulin biosynthesis and exocytosis machinery. We further performed Gene Ontology (GO) analysis of the biological processes. Consistent with KEGG pathway analysis, citrate metabolism, tricarboxylic acid (TCA) cycle, ɑ-amino acid metabolism, and FAO were among the top ten enriched processes (Fig. 2d), which are essential for β-cell function. Processes related to protein folding and vesicle-mediated transport were also enriched (Fig. 2d). This pattern suggests that islet acetylated proteins are involved in diverse insulin-secreting related functions.

### Identification of acetylated proteins in the context of metabolic signaling pathways for insulin secretion

**Fig. 3:**
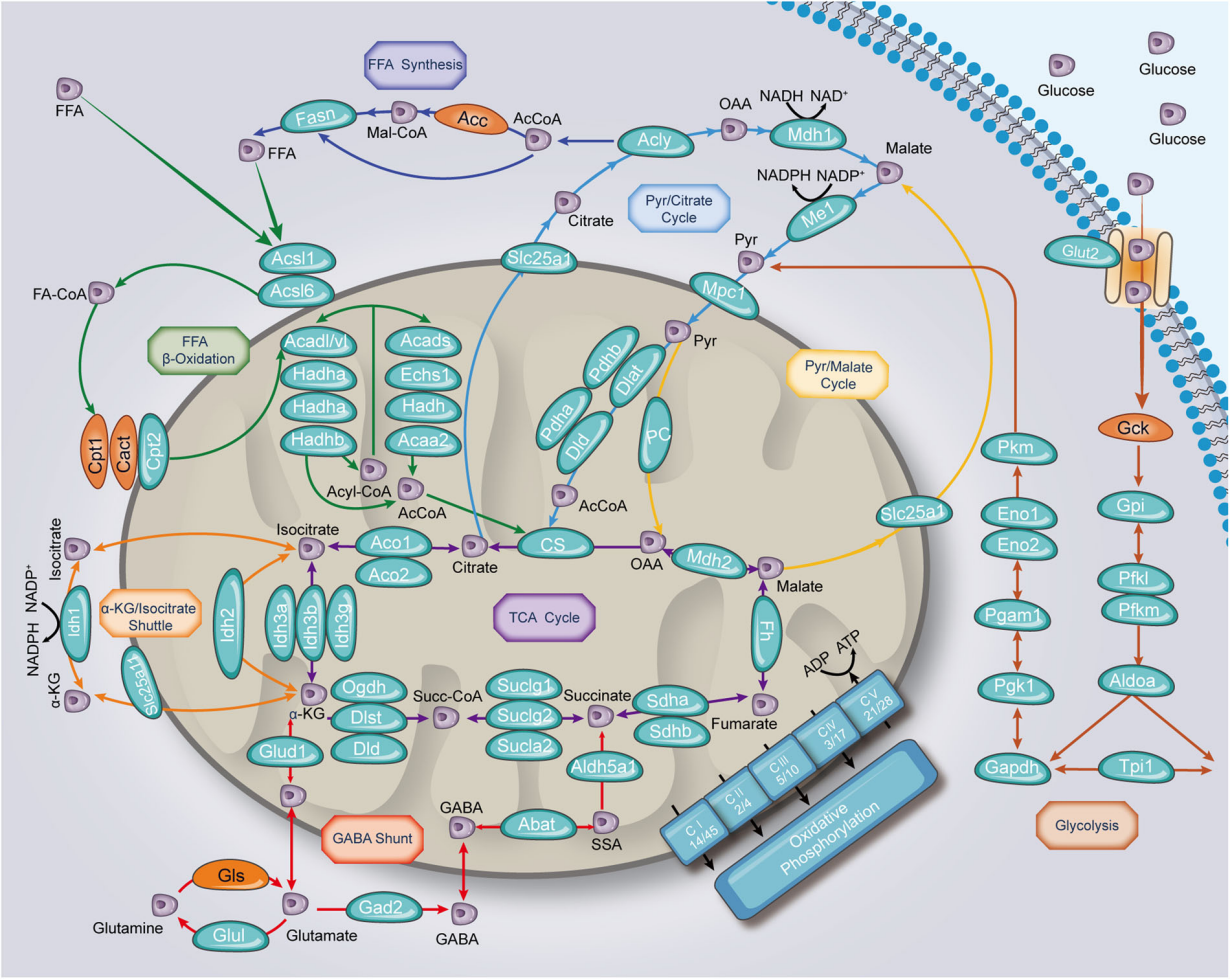
Acetylated proteins in the context of metabolic signaling pathways for insulin secretion. Proteins identified in the islet acetylome are labeled in bluish green and those not identified in orange. Arrows are color coded according to their metabolic processes

To gain an in-depth and integrated view of the correlation between acetylated proteins and β-cell function, we explored the coverage of our islet acetylome in the context of metabolic signalings for fuel-induced stimulus-secretion coupling^3^. As the main driver for insulin secretion, glucose undergoes glycolysis and is converted to pyruvate, which enters into mitochondria to participate in TCA cycle and oxidative phosphorylation, resulting in increased insulin exocytosis^23^. Almost every enzyme involved in the above processes was acetylated (Fig. 3).

This classical triggering process is complemented by amplification pathways and activation of auxiliary metabolic pathways^3,24^. Pyruvate in islets is converted to oxaloacetate via pyruvate carboxylase and further forms into citrate to enter TCA cycle or participate in pyruvate/citrate cycling, which is critical to modulate insulin secretion^25,26^. The involved enzymes (Pc, Slc25a1, Acly, and Mdh1) were all acetylated. Another pyruvate cycling is the pyruvate/malate cycle that exchanges malate to produce NADPH by Me1^27^, which could be acetylated. There also exists an isocitrate/α-KG shuttle in the control of GSIS^28^. The enzymes catalyzing this process (Idh1, Idh2, Idh3) have been identified in our acetylome (Fig. 3).

As to amino acid metabolism, glutamate gives rise to α-KG via Glud1 and GABA via Gad2, which further metabolized to succinate and enters TCA cycle. The involved enzymes in this GABA shunt are preeminent for anaplerosis to promote insulin secretion^3,29^, all of which were acetylated (Fig. 3).

Glucose metabolism is tightly linked to the production of lipid signaling molecules. Cellular free fatty acids (FFAs) could be synthesized by Fasn and further activated by Acsl, which were acetylated (Fig. 3). The generated FA-CoA either joins the glycerolipid/FFA (GL/FFA) cycle to promote insulin secretion, or diverts into mitochondria for β-oxidation^30^. Interestingly, as an off signal for insulin secretion, every enzyme involved in β-oxidation was found to be acetylated in islet (Fig. 3).

### Quantitative acetylome of rat islets in response to glucose

Islets pretreated with high glucose for 24 h secreted more insulin in response to 3.3, 16.7 mM glucose and KCl (Fig. S2A). Pretreatment with TSA and NAM showed a similar result (Fig. S2B). We further pretreated islets with TSA and NAM in the presence of either 1.4 or 5.6 mM glucose. Although 5.6 mM glucose alone potentiated GSIS compared with 1.4 mM glucose, GSIS was dramatically amplified by TSA and NAM pretreatment at 1.4 mM glucose (Fig. S2C), suggesting high glucose shared a similar pathway with acetylation to amplify insulin secretion.

**Fig. 4:**
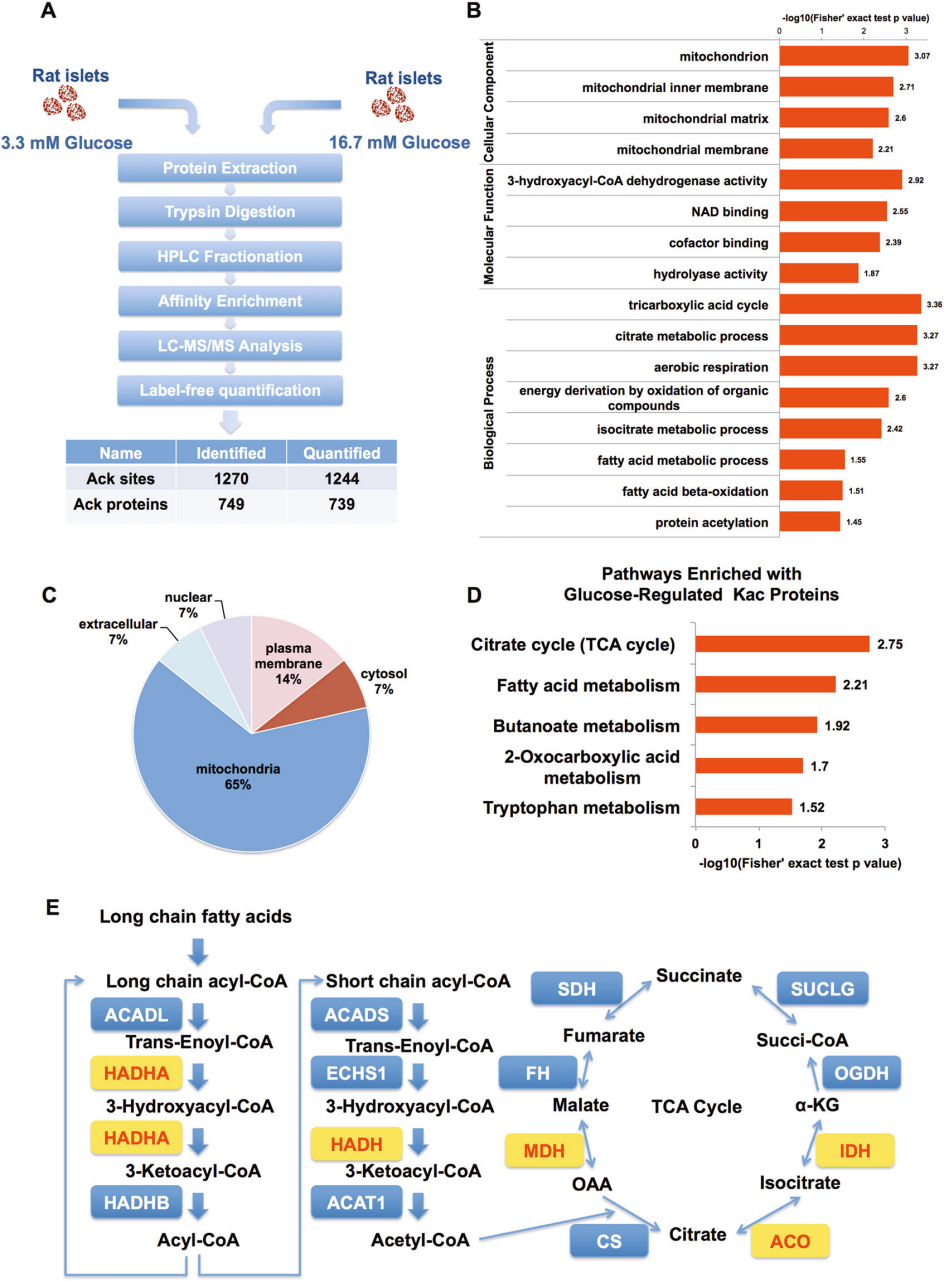
Quantitative acetylome of rat islets in response to glucose. **a** Proteomic workflow for the quantification of Kac sites and proteins in rat islets in response to low and high glucose. **b** GO-based enrichment analysis of glucose-regulated hyperacetylated proteins. **c** Subcellular distribution of glucose-regulated hyperacetylated proteins. **d** KEGG pathway-based enrichment analysis of glucose-regulated hyperacetylated proteins. **e** Mitochondrial fatty acid β-oxidation and TCA cycle enzymes (gene symbols) are highlighted next to the reaction they catalyze, with hyperacetylated enzymes targeted by high glucose colored in yellow. Logarithmized corrected *p*-values for significant overrepresentation are shown

To explore whether protein acetylation is linked to glucose-elicited metabolic flux involved in insulin secretion, we quantified the acetylomes of rat islets exposed to 3.3 and 16.7 mM glucose for 6 h. Using label-free quantitative proteomic approach, we identified 1270 lysine-acetylated sites in 749 proteins, among which 1244 acetylated sites in 739 proteins were accurately quantified in three parallel LC-MS/MS analyses (Fig. 4a). The quality of MS data is evaluated (Figure S1C and S1D). The fold-change cutoff was set to be 1.5. In high glucose-treated islets, the acetylation levels of 14 lysine sites in 14 proteins were significantly upregulated and those of 17 lysine sites in 16 proteins were significantly downregulated (Supplementary Table S2).

To understand the functions of glucose-controlled acetylated proteins, we divided the quantifiable acetylated proteins into four categories according to their fold-change ratios (Fig. S2D) and then plotted for KEGG enrichment-based cluster analysis. We found that the great majority of metabolic pathways were exclusively enriched with hyperacetylated proteins (Fig. S2E). Glucose-upregulated Kac proteins were more frequently present in mitochondria and exhibited 3-hydroxy-CoA dehydrogenase activity (Fig. 4b). The GO biological process analysis demonstrated that these proteins were involved in TCA cycle, aerobic respiration, and fatty acid metabolism (Fig. 4b). Among 14 upregulated Kac proteins, 9 were located in mitochondria, highlighting the importance of mitochondrial metabolism in glucose-modulated islet function (Fig. 4c).

Further KEGG pathway analysis found that many metabolic pathways were strongly influenced by glucose. The top pathways most significantly enriched with glucose-upregulated Kac targets were TCA cycle and fatty acid metabolism (Fig. 4d). It has been demonstrated that malonyl-CoA derived from glucose metabolism inhibits mitochondrial FAO in β-cells^31,32^. As multiple enzymes of FAO and TCA cycle were highly acetylated by glucose (Fig. 4e), it is possible that reversible protein acetylation is involved in glucose-modulated FAO.

### Glucose increases ECHA acetylation and decreases fatty acid β-oxidation of islets

**Fig. 5:**
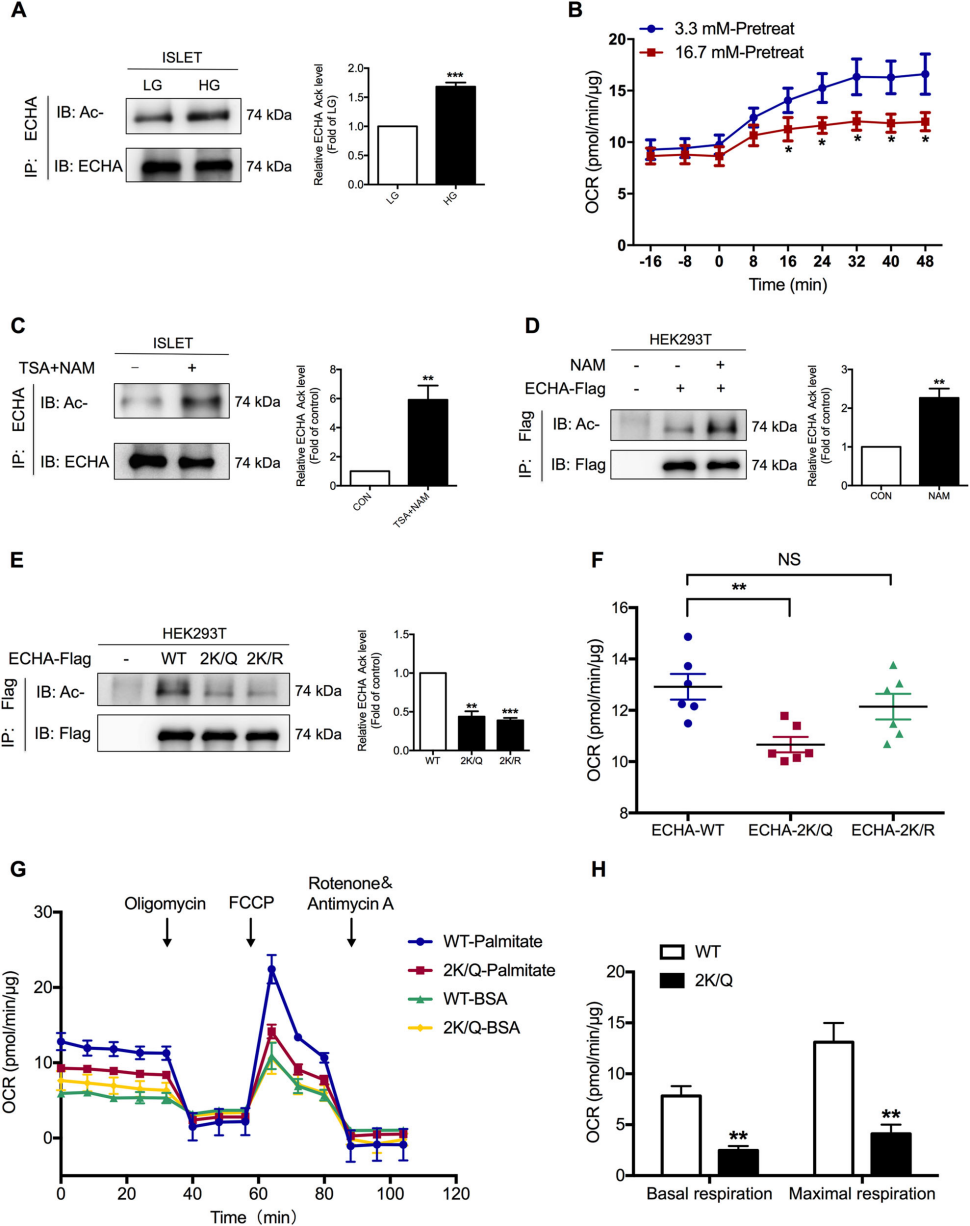
ECHA acetylation decreases fatty acid β-oxidation. **a** Acetylation levels of endogenous ECHA in rat islets treated with 3.3 mM (LG) and 16.7 mM glucose (HG). **b** Oxidation consumption rate (OCR) of palmitate was measured after rat islets were treated with 3.3 or 16.7 mM glucose for 6 h. **c** Acetylation levels of endogenous ECHA in rat islets treated with or without deacetylase inhibitors TSA plus NAM. **d** Detection of acetylation levels of ectopically expressed ECHA in HEK293T cells treated with or without NAM. **e** Flag-tagged wild type, K644K505Q (2 K/Q), and K644K505R (2 K/R) ECHA were overexpressed in HEK293T cells and acetylation levels were detected. **f** OCR of palmitate was measured after wild type, 2 K/Q, and 2 K/R ECHA were overexpressed in HEK293T cells. **g** Wild type and 2 K/Q ECHA were ectopically expressed in HEK293T cells and OCR was measured in the presence of palmitate–BSA or BSA control. Arrows indicate the time of addition for oligomycin, FCCP and rotenone/antimycin A. **h** Determination of basal and maximal respiration due to the utilization of exogenous palmitate in **g**. Data are expressed as mean ± SEM of at least three independent experiments. *p < 0.05, **p < 0.01, ***p < 0.001 vs. control (CON or WT)

Among glucose-upregulated acetylated enzymes in FAO, ECHA containing enoly-CoA hydratase and long-chain 3-hydroxyacyl-CoA dehydrogenase functions catalyzes the second and third step of long-chain fatty acid β-oxidation^33^. Glucose increased the acetylation levels of lysine residue K644 and K505 in ECHA (Fig. S2F). Rat islets treated with 16.7 mM glucose showed markedly induced ECHA acetylation, confirming the acetylome results (Fig. 5a). To investigate whether FAO in islets could be functionally impacted by glucose, we directly assessed OCR of palmitate under low glucose condition using Seahorse assay as a parameter of FAO. As previously reported^34^, 16.7 mM glucose pretreatment significantly decreased palmitate oxidation in rat islets compared with 3.3 mM glucose (Fig. 5b).

Similar to high glucose, TSA and NAM treatment also resulted in ECHA hyperacetylation of islets (Fig. 5c). Exogenously expressed ECHA in HEK293T cells was highly acetylated by NAM treatment (Fig. 5d). To determine whether the acetylation of ECHA K644 and K505 changes its ability to promote FAO, we further generated double glutamine (Q) or arginine (R) mutants of K644 and K505 (2 K/Q and 2 K/R). Both 2 K/Q and 2 K/R mutants resulted in significant decreases in ECHA acetylation (Fig. 5e). Moreover, the acetylation mimetic 2 K/Q mutant significantly decreased palmitate oxidation compared with wild-type ECHA, whereas the non-acetylable 2 K/R mutant hardly showed any difference (Fig. 5f). Then we explored the ability of 2 K/Q mutant to utilize exogenous fatty acids under mitochondrial stress (Fig. 5g). Both basal and maximal respiration rates due to utilization of exogenous palmitate were significantly decreased in cells expressed with 2 K/Q mutant (Fig. 5h), confirming that ECHA acetylation decreases FAO.

### SIRT3 deacetylates ECHA and increases fatty acid β-oxidation in β-cells

**Fig. 6:**
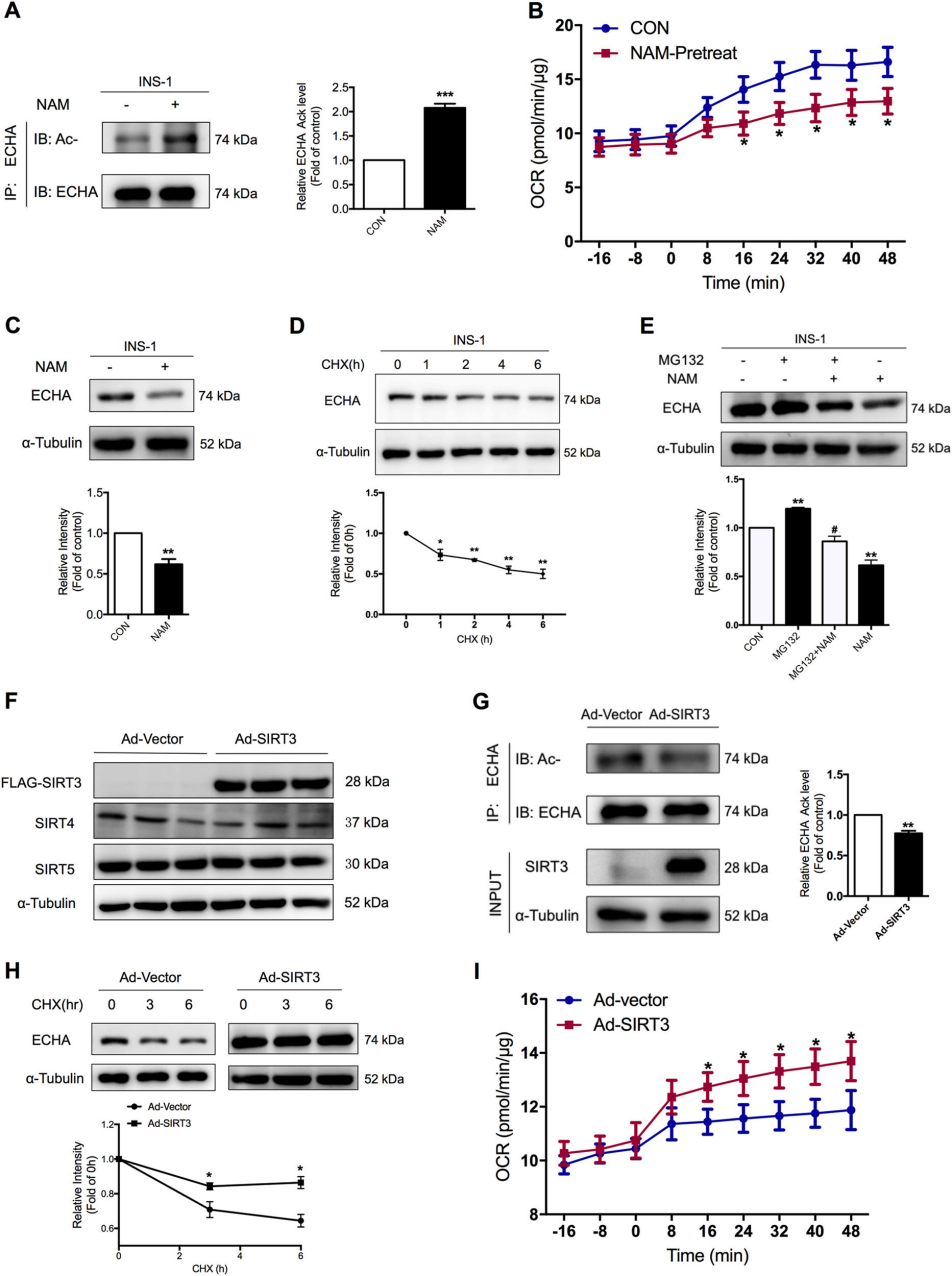
SIRT3 deacetylates ECHA and increases fatty acid β-oxidation in β cells. **a** Acetylation level of endogenous ECHA in INS-1 cells treated with or without 5 mM NAM for 6 h. **b** OCR of palmitate was measured after rat islets were treated with or without 5 mM NAM for 6 h. **c** ECHA protein level in INS-1 cells treated with or without 5 mM NAM for 6 h. **d** ECHA protein level in INS-1 cells treated with cycloheximide (CHX) for the indicated time. **e** After INS-1 cells were treated with or without NAM in the absence or presence of MG132, ECHA protein level was detected. **f** After rat islets were transfected with control vector (Ad-Vector) or SIRT3-overexpressing adenovirus (Ad-SIRT3), protein levels of SIRT3, SIRT4, and SIRT5 were detected. **g** After INS-1 cells were transfected with control vector or SIRT3-overexpressing adenovirus, cell lysates were immunoprecipitated with ECHA antibody and subjected to Western blot with anti-acetyllysine antibody. **h** INS-1 cells transfected with vector or SIRT3-overexpressing adenovirus were treated with cycloheximide for the indicated time and then ECHA protein level was detected. **i** Palmitate oxidation rate was measured after rat islets were transfected with control vector or SIRT3-overexpressing adenovirus. Data are expressed as mean ± SEM of three independent experiments. *p < 0.05, **p < 0.01, **p < 0.001 vs. control. #*p* < 0.05 vs. NAM

To confirm that ECHA was indeed acetylated in β-cells, we explored ECHA acetylation in a commonly used β-cell line INS-1 cells. ECHA was hyperacetylated in INS-1 cells treated with TSA plus NAM (Fig. S3A) or NAM alone (Fig. 6a). Similar to high glucose, palmitate oxidation was also decreased by NAM pretreatment (Fig. 6b). Furthermore, TSA and NAM co-treatment significantly reduced ECHA protein abundance in INS-1 cells (Fig. S3B), without changing its mRNA expression (Fig. S3C), suggesting that acetylation decreases protein stability of ECHA. Neither the acetylation (Fig. S3D) nor protein expression (Fig. S3E) levels of ECHA were changed by TSA. However, NAM treatment alone significantly decreased ECHA protein level (Fig. 6c), indicating ECHA is regulated only by sirtuins. After cycloheximide treatment, the protein level of ECHA was decreased over time (Fig. 6d). Treatment of MG132, a proteasome inhibitor, increased ECHA protein level and canceled the destabilization effect of NAM on ECHA (Fig. 6e), suggesting that acetylation-promoted degradation of ECHA is likely mediated by the proteasome pathway.

Of all the sirtuin members, SIRT3 is the only one with robust deacetylation activity^35^. ECHA has been reported to be a putative SIRT3 target in mice liver^36^. We wondered whether it was also regulated by SIRT3 in islet β-cells. After adenovirus-mediated SIRT3 overexpression, the acetylation abundance of several proteins was markedly reduced (Fig. S4A), without changing SIRT4 and SIRT5 mRNA and protein expressions (Figs. S4B and 6f). SIRT3 overexpression significantly decreased ECHA acetylation level (Fig. 6g) and prevented the degradation of ECHA protein in INS-1 cells (Fig. 6h). As SIRT3 promotes FAO in the liver^37,38^, we detected the impact of SIRT3 on FAO in islets. As expected, SIRT3 overexpression led to a substantial increase in palmitate oxidation rate of islets (Fig. 6i). Therefore, it is possible that SIRT3-controlled FAO flux is involved in fuel-mediated insulin secretion.

### Roles of SIRT3 and ECHA in regulating islet function

**Fig. 7:**
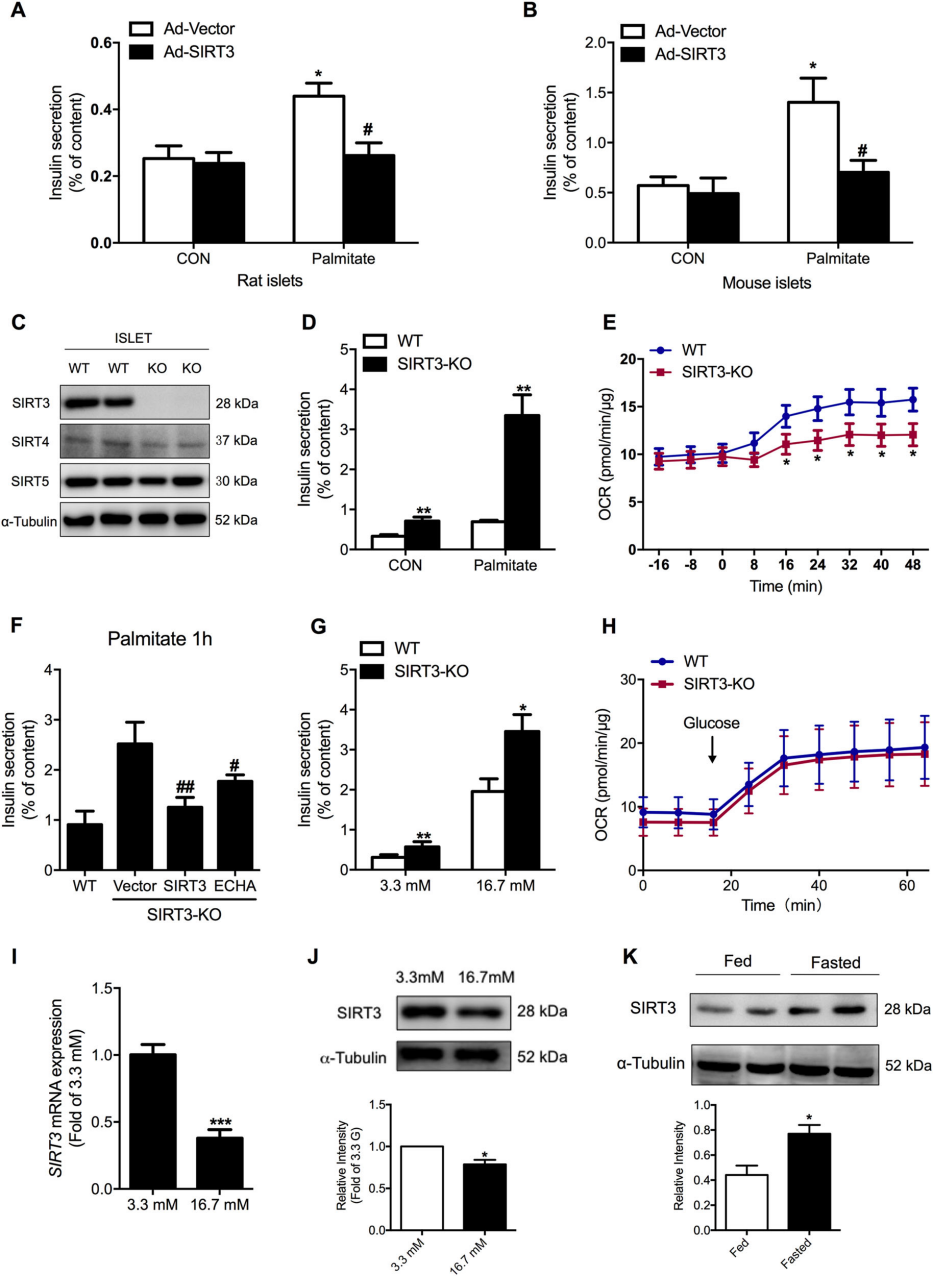
Role of SIRT3 in regulating islet function and metabolism. Rat islets (**a**) and mouse islets (**b**) transfected with control vector or SIRT3-overexpressing adenovirus were pretreated with 0.25 mM palmitate for 24 h and then stimulated with 3.3 mM glucose for insulin secretion assay. **c** Western blot analysis of SIRT3, SIRT4, and SIRT5 protein levels in islets isolated from wild type or SIRT3 knockout mice. **d** Islets isolated from wild type or SIRT3 knockout mice were stimulated with or without 0.25 mM palmitate at 3.3 mM glucose for 1 h and insulin secretion was assayed (*n* = 8). **e** Palmitate oxidation rate was measured in islets isolated from wild type or SIRT3 knockout mice (*n* = 5). **f** Islets from wild type or SIRT3 knockout mice were transfected with control vector and SIRT3 or ECHA-overexpressing adenovirus, and then stimulated with 0.25 mM palmitate for 1 h for insulin secretion assay. **g** Islets isolated from wild type or SIRT3 knockout mice were stimulated with 3.3 or 16.7 mM glucose for 1 h, and insulin secretion was assayed (*n* = 8). **h** OCR in islets isolated from wild type or SIRT3 knockout mice was measured at baseline or following glucose addition (*n* = 5). SIRT3 mRNA (**i**) and protein (**j**) expressions were detected after rat islets were treated with 3.3 or 16.7 mM glucose for 24 h. **k** Western blot analysis of SIRT3 protein levels in islets isolated from rats fed or fasted for 24 h. Data are expressed as mean ± SEM of three independent experiments. *p < 0.05, **p < 0.01, ***p < 0.001 vs. control (CON), WT, or 3.3 G. #*p* < 0.05, ##*p* < 0.01 vs. vector

As illustrated in Fig. 7a, insulin secretory response at basal glucose was significantly augmented by a 24 h palmitate pretreatment as shown previously^14^. SIRT3 overexpression decreased basal insulin hypersecretion in rat islets pretreated with palmitate (Fig. 7a). This was also the case in SIRT3-overexpressing mouse islets (Fig. 7b). We further used SIRT3 knockout mice to evaluate the ex vivo impact of SIRT3 on insulin secretion. Genotypes showed deletion of *SIRT3* gene (Fig. S4C) and western blotting confirmed knockout of SIRT3 protein in these mice (Fig. 7c). SIRT3 knockout mice showed no significant alterations in SIRT4 and SIRT5 expressions in both islets (Fig. 7c and S4D) and the liver (Fig. S4E). Palmitate-stimulated insulin secretion was markedly increased in SIRT3KO islets (Fig. 7d), which was reversed by SIRT3 overexpression (Fig. 7f). Consistent with the effect of NAM, SIRT3 knockout islets showed a significant decrease in palmitate oxidation rate (Fig. 7e). Taken together, these data highlight an important role of SIRT3 in regulating palmitate-stimulated insulin secretion.

To determine whether SIRT3 inhibited insulin secretion via deacetylating ECHA, rat islets were transfected with ECHA-overexpressing adenovirus (Fig. S5A). Both basal and high glucose-potentiated insulin release were significantly decreased in ECHA-overexpressing islets (Fig. S5B). ECHA overexpression markedly suppressed basal insulin hypersecretion induced by 16.7 mM glucose pretreatment (Fig. S5C). However, ECHA overexpression only partially reversed SIRT3 deficiency-stimulated insulin secretion under palmitate treatment (Fig. 7f).

Moreover, both basal and GSIS were significantly increased in SIRT3KO islets (Fig. 7g). We further explored mitochondrial glucose oxidation of SIRT3KO islets. Neither basal nor glucose oxidation showed any difference, indicating that increased insulin secretion of SIRT3KO islets is not attributed to glucose oxidation (Fig. 7h).

To determine SIRT3 expression in islets in response to nutrient change, rat islets were incubated for 24 h with 3.3 and 16.7 mM glucose. Both SIRT3 mRNA (Fig. 7i) and protein (Fig. 7j) expressions displayed significant decreases after high glucose treatment, in support for an increased mitochondrial acetylation status under this condition (Fig. 4c). We then observed SIRT3 expression in islets under fasting condition, which promotes FAO to suppress insulin secretion^39^. Consistent with the result of low glucose incubation, SIRT3 protein level was increased in islets of fasted rats (Fig. 7k).

## Discussion

Protein lysine acetylation represents an important mechanism to regulate overall energy metabolism^4^. In pancreatic islets, studies addressing the biological role of protein acetylation have been hampered by lacking knowledge of the modified targets. The present study performed the first comprehensive acetylation analysis in rat islets. Further islet quantitative acetylome revealed hyperacetylation of fatty acid β-oxidation enzymes under high glucose condition, which resulted in decreased FAO and enhanced β-cell function. Finally, we proposed a mechanism by which SIRT3 negatively regulates insulin secretion through stabilizing ECHA by deacetylation. These findings identify protein acetylation as a novel regulatory mechanism for insulin secretion.

The lysine acetylation profiles of many metabolic tissues highlighted the importance of protein acetylation in maintaining glucose homeostasis^7,9,11^. One study has provided an organ-wide map of lysine acetylation from 16 rat tissues and identified 2036 acetylated sites on 878 proteins in pancreas^9^. However, islets were not extracted from pancreas for this experiment. Interestingly, only partial acetylated sites and proteins we identified overlap with those in other tissues, suggesting a distinct pattern of acetylation in islets. We found of interest the broad coverage and highly abundance of the islet acetylome in metabolic pathways of β-cells related to nutrient sensing for insulin secretion (Fig. 3), emphasizing the role of acetylation in modulating tissue-specific function. As acetylation has the potential to affect enzyme activity, it is reasonable to suppose that acetylation-driven cooperativity of these metabolic pathways may be crucial to enhance β-cell function in the face of metabolic demand.

Pancreatic β-cells decrease insulin secretion during fasting to prevent hypoglycemia. It is widely accepted that elevated glucose inhibits FAO by decreasing AMPK activity and increasing malonyl-CoA production in β-cells, thus diverts FA-CoA into the GL/FFA cycle, providing “on” signals for insulin secretion^40^. On the contrary, fasting activates AMPK, enhances FAO, and “turns off” the switch for insulin secretion. The flux of FAO tightly links glucose and fatty acid metabolism to signaling for insulin secretion^14,30^. The present study revealed significantly increased SIRT3 expression in rat islets under low glucose and fasting status. It is reasonable to suppose that SIRT3 links the low energy status of islets to insulin hyposecretion. However, the physiological function of SIRT3 in β-cells remains largely obscure. It has been demonstrated that short hairpin RNA-mediated SIRT3 knockdown in MIN6 cells aggravated palmitate-induced dysfunction^41^, whereas SIRT3 overexpression partially recovered palmitate-impaired GSIS^42^. These in vitro studies only partially suppressed SIRT3 expression and assessed its function in the context of glucolipotoxicity. A recent study found that SIRT3 knockout in β-cell line had no effect on fuel-stimulated insulin secretion. In their islet perifusion experiments, islets of chow diet SIRT3KO mice showed an increased trend for insulin secretion in response to glucose and fatty acids, without statistical significance^43^. In our static incubation assay, primary islets of SIRT3KO mice secreted significantly more insulin compared with wild-type controls, in consistent with the results of Hirschey et al.^44^ that SIRT3KO mice revealed higher fasting and glucose-stimulated insulin levels. Therefore, the hyperinsulinemia of these mice may be attributed at least in part to SIRT3 deficiency caused insulin hypersecretion from β-cells, which accelerates the development of insulin resistance and metabolic syndrome. Moreover, we observed a marked decrease of palmitate-potentiated insulin secretion in SIRT3-overexpressing islets. In basal state, elevated plasma FFAs were responsible for some of the hyperinsulinemia in normoglycemic obese subjects^45^. Basal insulin levels are such an important determinant of insulin sensitivity that hyperinsulinemia initiates and contributes to insulin resistance in patients with obesity or type 2 diabetes^46^. Therefore, interfering this process by elevating SIRT3 expression or activity will protect β-cells against FFA-driven excess insulin secretion in these patients.

**Fig. 8:**
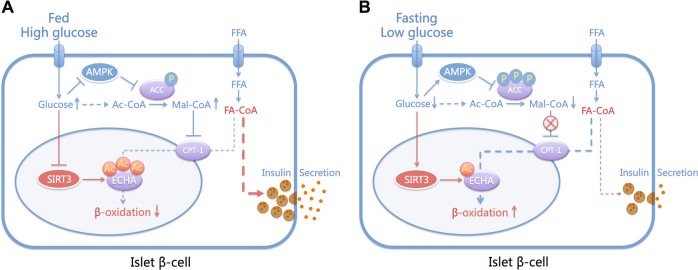
Role of the acetylation program in modulating insulin secretion in response to energy status. **a** Under fed condition, high glucose reduces AMPK activity, resulting in decreased phosphorylation of ACC and increased production of malonyl-CoA (Mal-CoA), which blocks fatty acyl-CoA (FA-CoA) entrance into mitochondria for β-oxidation via inhibiting CPT-1. On the other hand, high glucose decreases the expression or activity of SIRT3, leading to increased acetylation of ECHA and reduced mitochondrial β-oxidation. Thus, phosphorylation and acetylation programs cooperatively increase cytosolic lipid signaling molecules to promote insulin secretion in islet β-cell. **b** During fasting, activation of AMPK phosphorylates and inhibits ACC, resulting in reduced Mal-CoA and increased entry of substrates into mitochondria for β-oxidation. Low glucose also increases the expression or activity of SIRT3, which deacetylates ECHA and promotes fatty acid β-oxidation. The two signaling pathways cooperatively decrease lipid signaling molecules for insulin secretion

As a metabolic sensor, SIRT3 has been noted for its numerous roles in regulating mitochondrial biology^36,47^, especially fatty acid β-oxidation^38^. However, there is still no consensus as to its functional contribution to FAO, with both stimulatory and inhibitory effects observed^48^. Our study revealed that SIRT3 overexpression increased FAO rate, whereas SIRT3 knockout exhibited an opposite result in islets. The FAO enzymes ECHA and short-chain 3-hydroxyacyl-CoA dehydrogenase (SCHAD, coding gene *HADH*) were hyperacetylated by high glucose, and acetylation mimetic mutant of ECHA decreased FAO. SCHAD deficiency is associated with islet cell-autonomous hyperinsulinemic hypoglycemia^49^. Patients with long-chain 3-hydroxyacyl-CoA dehydrogenase deficiency caused by ECHA mutation also develop hypoglycemia^50^. To our knowledge, the effect of ECHA on islet function has never been reported. The present study showed that ECHA overexpression reduced basal and fuel-potentiated insulin secretion. Moreover, SIRT3 deacetylated ECHA and prevented its degradation. Apparently, SIRT3 couples the energy status of islets with insulin secretion via protein deacetylation. Under low fuel condition, AMPK-modulated activities of acetyl-CoA carboxylase (ACC) and carnitine palmitoyltransferase 1 (CPT-1) by phosphorylation determine the entry of FA-CoA into mitochondria for β-oxidation^3^ and SIRT3-mediated deacetylation of ECHA directly promotes FAO, in concert leading to decreased lipid signals for insulin secretion (Fig. 8). Therefore, SIRT3 exerts a new negative metabolic regulation of insulin secretion through a mechanism distinct from AMPK. Glucose decreases FAO flux and provides more lipid signals for insulin secretion, which may at least partially be attributed to the hyperacetylation of FAO enzymes.

In summary, this study provides a comprehensive picture of protein acetylation in rat islets and expands the inventory of known acetylated sites and proteins. The acetylation status of islet mitochondrial enzymes in response to nutrient change is crucial for insulin secretion via regulating FAO flux. As the mitochondrial deacetylase, SIRT3 reduces islet acetylation level of FAO enzymes via sensing energy status and promotes FAO, resulting in decreased lipid signaling molecules for insulin secretion. Therefore, modulating SIRT3-mediated acetylation status could be a new and promising approach to prevent the onset of hyperinsulinemia and insulin resistance.

## Supplementary information

Supplemental Material

Supplementary Table S1

Supplementary Table S2
