# Supplementary material for: The pivotal role of protein acetylation in linking glucose and fatty acid metabolism to β-cell function
Source: Cell Death Dis. 2019 Jan 25;10(2):66. doi: 10.1038/s41419-019-1349-z (PMC6347623; doi:10.1038/s41419-019-1349-z)
Supplement: Supplementary file 1 — Supplemental Material [file 41419_2019_1349_MOESM1_ESM.docx]

Figure S1. MS data evaluation. Mass error distributions of all the identified peptides (A) and peptide length distribution (B) in islet acetylome. Mass error distributions of all the identified peptides (C) and peptide length distribution (D) in quantitative label-free acetylome. The distribution of mass error was near zero and most of them were less than 0.02 Da, indicating that the mass accuracy of the MS data fits the requirement. The length of most peptides was distributed between 8 and 20, indicating that sample preparation reaches the standard.

Figure S2. Analysis of quantified Kac proteins in rat islets in response to glucose. (A) Rat islets were pretreated with 3.3 or 16.7 mM glucose for 24h, and then stimulated with 3.3, 16.7 mM glucose or 35 mM KCl for 1h. The supernatant was taken for insulin secretion assay. (B) Rat islets were pretreated with 200 nM TSA and 5 mM NAM, and then stimulated with 3.3, 16.7 mM glucose or 35 mM KCl for 1h for insulin secretion assay. (C) Rat islets were pretreated with 200 nM TSA and 5 mM NAM in the presence of 1.4 or 5.6 mM glucose, and then islets from each group were stimulated with 16.7 mM glucose for 1h for insulin secretion assay. (D) Distribution of quantified Kac proteins according to their fold-change ratios upon high glucose stimulation: Q1 (0< Ratio <1/1.5), Q2 (1/1.5< Ratio <1/1.2), Q3 (1.2 < Ratio <1.5) and Q4 (Ratio >1.5). (E) Heatmap from enrichment-based cluster analysis of KEGG pathways. (F) Quantification of ECHA K644 and K505 acetylation by label-free MS. **p*<0.05, ****p*<0.001 *vs* control (CON or LG).

Figure S3. ECHA acetylation and expression levels in INS-1 cells. (A) Acetylation and (B) protein levels of ECHA in INS-1 cells treated with or without TSA plus NAM. (C) mRNA expression of HADHA after TSA and NAM treatment in INS-1 cells. (D) Acetylation and (E) protein levels of ECHA in INS-1 cells treated with or without 200 nM TSA for 20h. Data are expressed as mean±SEM of three independent experiments.

Figure S4. Roles of SIRT3 overexpression and SIRT3 knockout. (A) INS-1 cell lysates were immunoblotted with anti-acetyllysine antibody after transfected with vector or SIRT3-overexpressing adenovirus. The arrows identify candidate SIRT3 targets. (B) mRNA expressions of SIRT3, SIRT4 and SIRT5 after INS-1 cells were transfected with control vector or SIRT3-overexpressing adenovirus. (C) Genotyping products of wild-type (WT) and SIRT3 knockout (KO) mice. Product sizes were 562 and 200bp for WT and KO alleles, respectively. (D) mRNA expressions of SIRT3, SIRT4 and SIRT5 in islets isolated from wildtype or SIRT3 knockout mice. (E) Western blot analysis of SIRT3, SIRT4 and SIRT5 protein levels in livers of wildtype or SIRT3 knockout mice. Data are expressed as mean±SEM of three independent experiments. ***p*<0.01 *vs* vector control.

Figure S5. Role of ECHA in insulin secretion. (A) ECHA protein expression in rat islets transfected with vector and ECHA overexpressing adenovirus. (B) Rat islets transfected with vector and ECHA overexpressing adenovirus were treated with 3.3 and 16.7 mM glucose for 6h, and insulin secretion was measured. (C) Rat islets transfected with vector and ECHA overexpressing adenovirus were pretreated with 3.3 and 16.7 mM glucose for 24h, and then stimulated with 5.6mM glucose for 1h for insulin secretion assay. **p*<0.05, ***p*<0.01 *vs* vector control.

**Supplemental material and methods**

**Mass spectrometry and data analysis**

Protein extraction

Pooled islet sample was first grinded by liquid nitrogen, then the cell powder was transferred to 5 mL centrifuge tube and sonicated three times on ice using a high intensity ultrasonic processor (Scientz) in lysis buffer (8 M urea, 10 mM DTT, 2 mM EDTA, 3 μM TSA, 50 mM NAM and 1% protease inhibitor Cocktail Ⅲ). The remaining debris was removed by centrifugation at 20,000g at 4 °C for 10 min. Finally, protein was precipitated with cold 15% TCA for 2 h at -20 °C. After centrifugation at 4 °C for 10 min, supernatant was discarded. The remaining precipitate was washed with cold acetone for three times. Lysate was redissolved in buffer (8 M urea, 100 mM NH_4_CO_3_, pH 8.0) and protein concentration was determined with 2-D Quant kit according to the manufacturer’s instructions.

Trypsin digestion

For digestion, the protein solution was reduced with 10 mM DTT for 1 h at 37 °C and alkylated with 20 mM IAA for 45 min at room temperature in darkness. For trypsin digestion, the protein sample was diluted by adding 100 mM NH_4_CO_3_ to urea concentration less than 2M. Finally, trypsin (Promega) was added at 1:50 trypsin-to-protein mass ratio for the first digestion overnight and 1:100 trypsin-to-protein mass ratio for a second 4 h-digestion.

HPLC fractionation

Fractionation was then carried out by high pH reverse-phase HPLC using Agilent 300 Extend C18 column (5 μm particles, 4.6 mm ID, 250 mm length). Briefly, peptides were firstly separated with a gradient of 2% to 60% acetonitrile in 10 mM ammonium bicarbonate pH 10 over 80 min into 80 fractions. Then, the peptides were combined into 5 fractions and dried by vacuum centrifuging.

Affinity enrichment

To enrich Kac peptides, tryptic peptides dissolved in NETN buffer (100 mM NaCl, 1 mM EDTA, 50 mM Tris-HCl, 0.5% NP-40, pH 8.0) were incubated with pan-acetyl lysine antibody beads (PTM Biolabs) at 4°C overnight with gentle shaking. The beads were washed four times with NETN buffer and twice with ddH_2_O. The bound peptides were eluted from the beads with 0.1% TFA. The eluted fractions were combined and vacuum-dried. The resulting peptides were cleaned with C18 ZipTips (Millipore) according to the manufacturer’s instructions, followed by LC-MS/MS analysis.

LC-MS/MS analysis

Three parallel analyses for each fraction were performed. Peptides were dissolved in 0.1% formic acid, directly loaded onto a reversed-phase pre-column (Acclaim PepMap 100, Thermo Scientific). Peptide separation was performed using a reversed-phase analytical column (Acclaim PepMap RSLC, Thermo Scientific). The gradient was comprised of an increase from 6% to 22% solvent B (0.1% FA in 98% ACN) for 24 min, 22 % to 35% for 8 min and climbing to 80% in 5 min then holding at 80% for the last 3 min, all at a constant flow rate of 300 nl/min on an EASY-nLC 1000 UPLC system, the resulting peptides were analyzed by Q Exactive^TM^ Plus hybrid quadrupole-Orbitrap mass spectrometer (Thermo Scientific).

The peptides were subjected to NSI source followed by tandem mass spectrometry (MS/MS) in Q Exactive^TM^ Plus (Thermo) coupled online to the UPLC. Intact peptides were detected in the Orbitrap at a resolution of 70,000. Peptides were selected for MS/MS using NCE setting as 30; ion fragments were detected in the Orbitrap at a resolution of 17,500. A data-dependent procedure that alternated between one MS scan followed by 20 MS/MS scans was applied for the top 20 precursor ions above a threshold ion count of 10000 in the MS survey scan with 15.0s dynamic exclusion. The electrospray voltage applied was 2.0 kV. Automatic gain control (AGC) was used to prevent overfilling of the ion trap; 5E4 ions were accumulated for generation of MS/MS spectra. For MS scans, the m/z scan range was 350 to 1800.

Database searching

The resulting MS/MS data was processed using MaxQuant with integrated Andromeda search engine (v.1.4.2). Tandem mass spectra were searched against *UniProt_rat* database concatenated with reverse decoy database. Trypsin/P was specified as cleavage enzyme allowing up to 4 missing cleavages, 5 modifications per peptide and 5 charges. Mass error was set to 10 ppm for precursor ions and 0.02 Da for fragment ions. Carbamidomethylation on Cys was specified as fixed modification and oxidation on Met, acetylation on Lys and acetylation on protein N-terminal were specified as variable modifications. Minimum peptide length was set at 7. All the other parameters in MaxQuant were set to default values. The site localization probability was set as > 0.75. Label-free quantification was performed with the LFQ algorithm in MaxQuant. FDR was adjusted to < 1% and minimum score for modified peptides score was set ≥ 40. Three biological replicates were grouped for quantification analysis. The ANOVA statistical test was applied to data from the three biological replicates, and Kac peptides with significant changes were identified (*P*< 0.05). We also applied a minimum 1.5-fold change cutoff to the list of regulated Kac peptides to identify proteins with differential regulation.

**Bioinformatic analyses**

Protein annotation

Identified proteins were annotated for Gene Ontology (GO) from UniProt-GOA database and were then classified based on three categories: biological process, cellular component and molecular function. Pathway annotation was performed using Kyoto Encyclopedia of Genes and Genomes (KEGG) database. Wolfpsort software was used to predict protein subcellular localization.

Functional enrichment

Functional annotation tool of the DAVID bioinformatics resource was used to identify enriched GO categories, KEGG pathways and protein domains. A two-tailed Fisher’s exact test was employed to test the enrichment of the annotated terms against a background including all rat proteins.

Enrichment-based clustering

We first collated all the protein groups obtained after functional enrichment analysis along with their p values, and then filtered for those categories which were at least enriched in one of the protein groups with p value <0.05. The corrected p values were converted to its Log value using P=-Log_10_ (p-value) and then transformed to z-score. For each category, the annotation was clustered based on z-score using one-way hierarchical clustering (Euclidean distance, average linkage clustering). Cluster membership was visualized by a heat map using the “heatmap.2” function from the “gplots” R-package.
